# Methods for home-based self-applied polysomnography: the Multicenter AIDS Cohort Study

**DOI:** 10.1093/sleepadvances/zpac011

**Published:** 2022-04-29

**Authors:** Naresh M Punjabi, Todd Brown, R Nisha Aurora, Sanjay R Patel, Valentina Stosor, Joshua Hyong-Jin Cho, Halla Helgadóttir, Jón Skírnir Ágústsson, Gypsyamber D’Souza, Joseph B Margolick

**Affiliations:** Division of Pulmonary, Critical Care, and Sleep Medicine, University of Miami, Miller School of Medicine, Coral Gables, FL, USA; Division of Endocrinology, Diabetes, & Metabolism, Johns Hopkins University, School of Medicine, Baltimore, MD, USA; Division of Pulmonary and Critical Care Medicine, Robert Wood Johnson University Hospital, New Brunswick, NJ, USA; Pulmonary, Allergy, and Critical Care Medicine, University of Pittsburgh, School of Medicine, Pittsburgh, PA, USA; Department of Medicine, Northwestern University Feinberg School of Medicine, Chicago, IL, USA; Department of Psychiatry and Biobehavioral Sciences, University of California (Los Angeles), David Geffen School of Medicine, Los Angeles, CA, USA; Nox Medical, Reykjavík, Iceland; Nox Medical, Reykjavík, Iceland; Department of Molecular Microbiology and Immunology, Johns Hopkins University, Bloomberg School of Public Health, Baltimore, MD, USA; Department of Molecular Microbiology and Immunology, Johns Hopkins University, Bloomberg School of Public Health, Baltimore, MD, USA

**Keywords:** home testing, epidemiology, instrumentation

## Abstract

**Study Objectives:**

Along with multiple chronic comorbidities, sleep disorders are prevalent in people living with human immunodeficiency virus (HIV) infection. The goal of this study was to establish methods for assessing sleep quality and breathing-related disorders using self-applied home polysomnography in people with and without HIV.

**Methods:**

Self-applied polysomnography was conducted on 960 participants in the Multicenter AIDS Cohort Study (MACS) using the Nox A1 recorder to collect data on the frontal electroencephalogram (EEG), bilateral electrooculograms, and a frontalis electromyogram during sleep. Breathing patterns were characterized using respiratory inductance plethysmography bands and pulse oximetry. Continuous recordings of the electrocardiogram were also obtained. All studies were scored centrally for sleep stages and disordered breathing events.

**Results:**

Successful home polysomnography was obtained in 807 of 960 participants on the first attempt and 44 participants on the second. Thus, a successful polysomnogram was obtained in 851 (88.6%) of the participants. Reasons for an unsuccessful study included less than 3 h of data on oximetry (34.6%), EEG (28.4%), respiratory inductance plethysmography (21.0%), or two or more of these combined (16.0%). Of the successful studies (*N* = 851), signal quality was rated as good, very good, or excellent in 810 (95.2%). No temporal trends in study quality were noted. Independent correlates of an unsuccessful study included black race, current smoking, and cocaine use.

**Conclusions:**

Home polysomnography was successfully completed in the MACS demonstrating its feasibility in a community cohort. Given the burden of in-lab polysomnography, the methods described herein provide a cost-effective alternative for collecting sleep data in the home.

**Statement of Significance**

This study demonstrates the feasibility and success of conducting self-applied full montage polysomnography at home in a large community cohort. Using the methods described for conducting unattended home polysomnography, data needed to link sleep-related abnormalities to incident cardiovascular and noncardiovascular outcomes in HIV infection were collected.

## Introduction

Sleepiness and fatigue are recognized as major sources of morbidity in people living with human immunodeficiency virus (HIV) infection [1, 2] and can significantly impair functional status [3] and cognition [4]. Approximately 60% of people living with HIV have some form of sleep disturbance, the most common being sleep maintenance insomnia [2]. Habitually long sleep duration [5], restless legs syndrome [6], and obstructive sleep apnea [7] have also been reported in people living with HIV. The use of nonnucleoside reverse transcriptase inhibitors has been associated with lipohypertrophy (i.e. increase in central fat deposition) and lipoatrophy (i.e. decrease in peripheral fat) which can predispose to obstructive sleep apnea [8]. Abnormalities in sleep architecture and poor sleep quality have also been previously documented in early stage HIV infection prior to the development of acquired immunodeficiency syndrome (AIDS) [9, 10]. Altered sleep architecture in people living with HIV has been attributed to increased circulating levels of somnogenic cytokines, such as tumor necrosis factor-α and interleukin-1β [3, 9].

Despite the increase in clinical recognition, people living with HIV is less likely to be diagnosed with sleep disorders than people without [11]. Moreover, the longitudinal impact of sleep disorders on HIV-related comorbidity is not known. Thus, to advance the understanding of the burden and health implications of sleep disorders in people living with HIV, assessment of sleep was conducted in the Multicenter AIDS Cohort Study (MACS) [12]. A unique aspect of the data collection was the use of self-applied home polysomnography which included a novel frontal montage for recording the sleep electroencephalogram (EEG). In the current report, the development and implementation of methods for collecting, processing, and analyzing home self-applied polysomnography data are described, along with participant characteristics correlated with being able to provide a successful self-applied polysomnogram. These methods are unique in that they provide a robust methodology for collecting sleep data for other community-based studies that can then helpfully assess the potential associations between sleep health and other chronic disease conditions.

## Methods

### Study population

The MACS is an ongoing cohort study of the natural and treated history of HIV infection in men who have sex with men conducted in Baltimore/Washington DC, Chicago, Pittsburgh/Ohio, and Los Angeles [12]. The MACS, which includes both HIV-infected and HIV-uninfected men, was initiated in 1984 and has had four waves of enrollment (1984–1985, 1987–1991, 2001–2003, and 2010–2017). Study participants undergo semiannual visits for standardized interviews which include assessment of health behaviors (e.g. smoking status, use of recreational drugs and alcohol), physical examination including anthropometry, and collection of blood and urine specimens for laboratory testing and storage. From March 2018 to June 2019, all active MACS participants were invited to enroll for assessment of sleep as described herein. Informed consent was obtained from study participants and the protocol was approved by an Institutional Review Board at each of the study sites.

### Home self-applied polysomnography

The home sleep study was conducted using a self-applied portable sleep recorder (Nox A1, Nox Medical, Rejkjavik, Iceland). The Nox A1 recorder has the ability to collect a frontal EEG montage using an optimized cable design for self-application. The potential concern of the burden incurred by recording the EEG monitoring during sleep at home was addressed prior to the initiation of the study by the development of standardized procedures and participant instructions for securing the self-applied electrodes including those for the customized EEG cable. Using this cable, the Nox A1 recorder can be used to record the right and left electrooculograms (EOG), a frontal EEG montage (AF4, AF3, AF7, and AF8), the frontalis muscle electromyogram (EMG), the electrocardiogram (ECG), nasal airflow, oxygen saturation, chest and abdominal respiratory inductance plethysmography bands, and right and left anterior tibialis EMG. The Nox A1 recorder is powered with one AA battery and can record the aforementioned signals for approximately 8–10 h.

Prior to each participant’s semiannual study visit, a package was prepared for each participant (see online supplement for manual of procedures). This included the following: (1) the Nox A1 recorder with the necessary attachments including reusable EEG, ECG, and leg EMG cables; (2) a wireless Nonin oximeter, which uses a bluetooth connection to the Nox A1 recorder; and (3) nonreusable items including the EEG, ECG, and EMG electrodes; a nasal cannula for assessments of airflow; and respiratory inductance plethysmography bands for recording of chest and abdominal movements. During the clinic visit, the participant was trained on the use of the Nox A1 recorder, and the preferred bed time and wake time were ascertained to configure the start and stop time for data collection for one night of recording. Instructional videos were developed along with notecards placed in the package to help participants complete the hookup of the Nox A1 recorder at home without the assistance of an onsite technician.

### Participant instruction

The study protocol required that study staff explain to study participants procedures for the proper use of the Nox A1 recorder. Accordingly, a video illustrating these procedures was prepared and initially piloted by study staff. After several iterations, the final video was approximately 15 min long, and research staff was able to put on the Nox A1 recorder and all associated cables and attachments in approximately 20 min. We also assessed the ability of study staff to perform the overnight sleep study on themselves and to train study participants to do the same, including putting on the Nox A1 recorder. After this was successfully demonstrated for at least two studies for each staff member, the clinic staff at that site were certified to train study participants, and the study protocol and training materials were implemented at the clinical sites. To this end, the purpose of the sleep study was explained to study participants during their regular semiannual MACS study visits. For those who agreed to participate in the sleep assessments, the procedures for using the Nox A1 recorder overnight were explained in detail, and each participant was given a sleep study package, as described above, that contained all necessary equipment and materials for conducting the study at home. They were also given a URL to access the online video illustrating application of the Nox A1 recorder and associated equipment, and a telephone number at which sleep study staff would be available in case assistance was needed. Participant instructions are available online as supplementary documents. The instructional video is available from the authors.

### Download, transfer, and review of sleep data

After completion of the overnight sleep study, the Nox A1 recorder was returned to the local study staff by overnight mail using a prepaid mailer or by delivering it to the clinic. Study staff at each of the study sites then downloaded the data from the Nox A1 recorder to a computer and transmitted the data to the central reading facility, where the quality of each sleep study was assessed based on the duration of the overall recording and duration of each recorded channel. A home sleep study was considered successful if all signals were present for at least 3 h. Successful studies were further rated as excellent, very good, good, or fair based on the duration of artifact-free data for the various signals. After the initial review, each sleep study was manually scored for sleep staging using standard criteria [13]. Sleep staging for wakefulness, N1, N2, N3, and rapid eye movement sleep (REM) was conducted at 30-s intervals. Because a chin EMG electrode was not used, the frontalis muscle electrode was used to identify changes in EMG activity. For the scoring of disordered breathing events, apneas were identified if airflow was absent or nearly absent for at least 10 s. Apneas were classified as obstructive if chest or abdominal wall movement was noted, or central if no abdominal and chest wall movement was observed. Hypopneas were identified when there was a reduction of 30% or more in airflow for at least 10 s, and a 3% decrease in oxygen saturation. An alternative, more stringent definition was also used, which required a 4% decrease in oxygen saturation. The apnea–hypopnea index (AHI) was defined as the number of apneas and hypopneas per hour of sleep. Arousals were identified by standard criteria [13], and the arousal index was defined as the number of arousals per hour of sleep. To assess interscorer reliability of sleep staging and scoring of respiratory events, approximately 5% of the sleep studies were randomly selected and rescored by a different scorer.

### Statistical analysis

To assess the quality of data collected, the proportion of successful home sleep studies was assessed collectively for all sites and then for individual sites over the 14 months of data collection. Descriptive analysis for the primary cause of an unsuccessful home sleep study was conducted using frequency distributions. Interscorer reliability of scoring sleep stages, AHI, and arousal indices were estimated using the intraclass correlation coefficient (ICC). Differences by study sites were compared by analysis of variance (ANOVA). To examine associations between participants’ characteristics and their completion of a successful home polysomnogram, multivariable regression analyses were utilized. All analyses were conducted in SAS 9.0 (SAS Institute, Cary, NC), and statistical significance was set at an *α* value of 0.05.

## Results

### Feasibility and success rate

**Figure 1.**
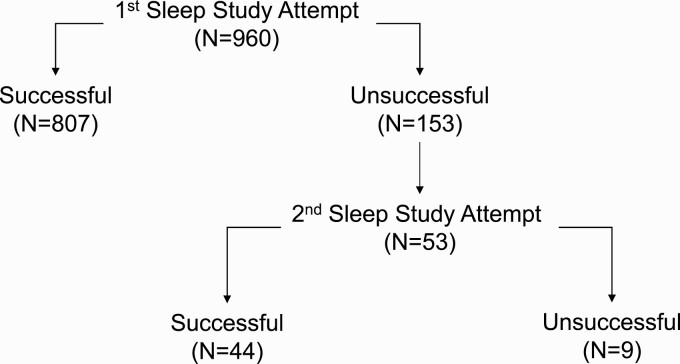
Numbers of study participants with successful and unsuccessful home sleep studies.

**Figure 2.**
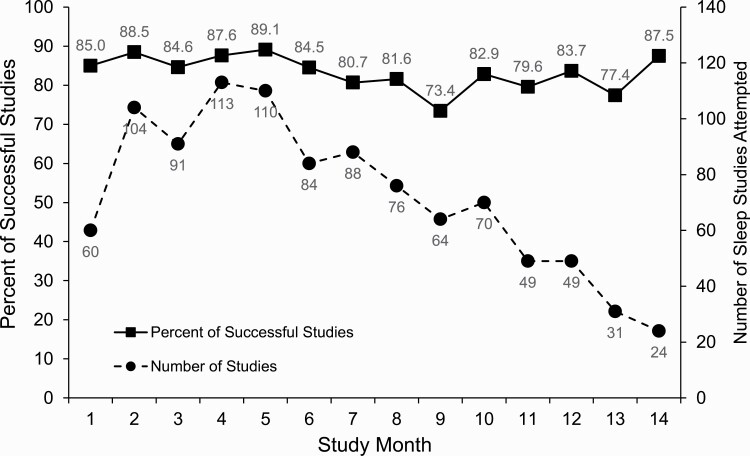
Numbers of home sleep studies conducted and proportions successful by study month.

A total of 960 MACS participants performed overnight in-home self-applied polysomnography. Of these, 807 (84.1%) provided a successful overnight polysomnogram on the first attempt. Of the remaining 153 participants (15.9%), 53 made a second attempt, and 44 (85.3%) of these yielded successful studies (Figure 1). Thus, 851 (88.6%) of the 960 study participants provided a successful sleep study. Overall, of 1013 home sleep studies performed, 851 (84.0%) were successful, and 162 (16.0%) were not. Reasons for the latter are shown in Table 1 for the full sample and by study site. The most common reasons for an unsuccessful study were less than 3 h of oximetry data (34.6%) and less than 3 h of EEG data (28.4%). Less than 3 h of data from respiratory inductance plethysmography bands or from multiple signals accounted for 21.0% and 16.0% of the unsuccessful studies, respectively. Figure 2 displays the proportions of successful and unsuccessful studies across all sites by study month. No temporal trends were observed in these proportions over the 14 months either in the full sample (Figure 2) or at any study site (Supplementary Figure S1). Temporal trends in unsuccessful studies due to either EEG or respiratory signals were also not observed in the aggregate sample or by site (Supplementary Table S1).

**Table 1.** Numbers of participants and of sleep studies performed, and reasons for unsuccessful studies

	Number (%)				
	All sites	Site 1	Site 2	Site 3	Site 4
Study participants	960	276	201	233	250
Number of studies	1013	288	214	239	272
Number (%) unsuccessful	162 (16.0)	45 (15.6)	25 (11.7)	40 (16.7)	52 (19.1)
Reason for unsuccessful study					
Oximetry <3 h	56 (34.6)	15 (33.3)	11 (44.0)	11 (27.5)	19 (36.6)
EEG* <3 h	46 (28.4)	10 (22.2)	6 (24.0)	13 (32.5)	17 (32.7)
Chest–abdomen <3 h	34 (21.0)	8 (17.8)	5 (20.0)	11 (27.5)	10 (19.2)
Two or more signals <3 h	26 (16.0)	12 (26.7)	3 (12.0)	5 (12.5)	6 (11.5)

*Electroencephalogram.

### Participant characteristics associated with a successful study

Table 2 shows characteristics of men who provided a successful sleep study (*n* = 851) and those who did not (*n* = 109). Compared to the former, the latter were younger, more likely to be nonwhite and current smokers, and less likely to have education beyond high school. They also had lower income levels and had greater alcohol and cocaine use. Multivariable logistic regression was used to examine which of the aforementioned factors were independently associated with a successful versus an unsuccessful study (Table 3). After adjusting for age, body mass index (BMI), center, education, and income, provision of a successful study was significantly associated only with race, smoking status, and cocaine use. Specifically, blacks were less likely than whites to complete a successful home sleep study (adjusted odds ratio: 0.34; 95% confidence interval [CI]: 0.18–0.65). Men who were multiracial or of another race also had a lower odds ratio (adjusted odds ratio: 0.80; 95% CI: 0.21–3.01) in a successful study, although these were not statistically different than that for white men. Furthermore, men who currently smoked cigarettes or used cocaine were also less likely to complete a successful study (adjusted odds ratio: 0.40; 95% CI: 0.20–0.81 and 0.40; 95% CI: 0.20–0.81, respectively). In sensitivity analyses, inclusion in the models of other characteristics such as HIV serostatus or prevalent hypertension, renal disease, or cardiovascular disease did not materially alter these results.

**Table 2.** Characteristics of participants with a successful or unsuccessful home sleep study

	Successful participants (*N* = 851)	Unsuccessful participants (*N* = 109)	*p* *
Age, years	57.5 (11.6)	53.9 (11.7)	0.003
BMI, kg/m^2^	27.5 (5.2)	26.7 (5.0)	0.15
Race			
White	551 (64.8%)	56 (51.3%)	<0.001
Black	214 (25.2%)	42 (38.5%)	
Other	42 (4.9%)	8 (7.3%)	
Multiracial	43 (5.1%)	3 (2.8%)	
Missing data	1 (0.1%)	0 (0.0%)	
Ethnicity			0.93
Hispanic	112 (13.2%)	14 (12.8%)	
Non-Hispanic	739 (86.8%)	95 (87.2%)	
Education			
≤11th grade	43 (5.0%)	11 (10.1%)	<0.001
12th grade	102 (12.0%)	29 (26.6%)	
Some college	211 (24.8%)	26 (23.8%)	
College degree	178 (20.9%)	19 (17.4%)	
Graduate work	296 (34.8%)	21 (19.3%)	
Missing data	21 (2.5%)	3 (2.8%)	
Income, $			
<10 000	124 (14.6%)	27 (24.8%)	0.005
10 000–19 000	104 (12.2%)	22 (20.2%)	
20 000–39 999	147 (17.3%)	18 (16.5%)	
40 000–59 999	144 (16.9%)	9 (8.3%)	
60 000–99 999	128 (15.0%)	11 (10.0%)	
≥100 000	132 (15.5%)	12 (11.0%)	
Missing data	72 (8.5%)	10 (9.2%)	
Smoking			
Never	279 (32.8%)	23 (21.1%)	<0.001
Former	402 (47.2%)	40 (36.7%)	
Current	149 (17.5%)	43 (39.4%)	
Missing data	21 (2.5%)	3 (2.8%)	
Alcohol, drinks/week			
None	180 (21.4%)	33 (30.3%)	0.031
1–3	407 (47.8%)	44 (40.4%)	
≥4	238 (28.0%)	25 (22.9%)	
Missing data	26 (3.1)	7 (6.4%)	
Cocaine use			
No	755 (88.7%)	81 (74.3%)	<0.001
Yes	64 (7.5%)	21 (19.3%)	
Missing data	32 (3.8%)	77 (6.4%)	
HIV seropositive	474 (55.7%)	68 (62.4%)	0.19

*p values computed with *t*-test for continuous variables and *χ*^2^ for categorical variables.

**Table 3.** Adjusted odds ratios for a successful home sleep study

Parameter	Odds ratio	95% CI
Race		
White	Reference	
Black	0.34	0.18–0.65
Other	0.48	0.17–1.41
Multiracial	0.80	0.21–3.01
Cigarette smoking		
Never	Reference	
Former	0.88	0.48–1.63
Current	0.40	0.20–0.81
Cocaine use	0.47	0.24–0.93

Adjusted odds ratio derived from a multivariable logistic regression model with age, BMI, center, race, cocaine use (yes or no), cigarette smoking (never, former, current), income level, and education level.

### Overall quality of successful studies

**Figure 3.**
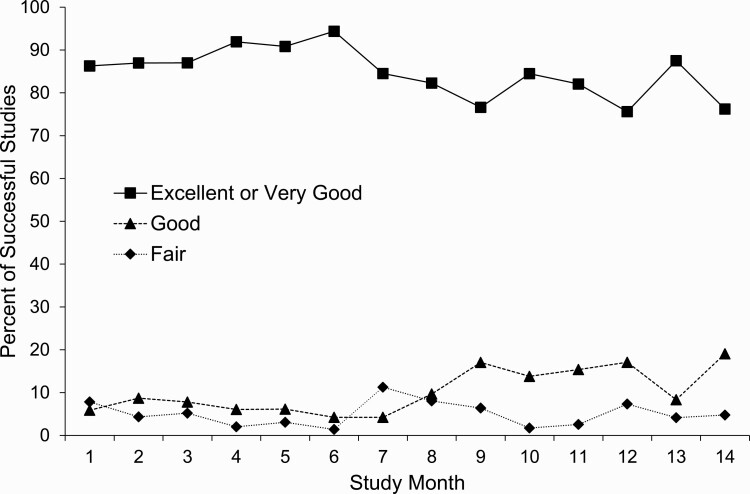
Numbers of home sleep studies completed and proportions of studies deemed successful by study month.

Among the 851 successful studies, there was some variation in quality by site (Table 4), but this was not significant (*p* = 0.25 for between-site differences by ANOVA). Overall, 72.4% of the studies were excellent and an additional 13.9% were very good, for a total of 86.3% of studies classified as excellent or very good (ranging from 84.0% to 88.4% across the sites). More than 93% of the 851 successful studies were rated either good, very good, or excellent, with no temporal trends in study quality either for the full study (Figure 3) or within specific sites (Supplementary Figure S2). Signal quality was best for chest and abdomen respiratory inductance plethysmography bands, and lowest for the nasal pressure and ECG signals, albeit still very good in the vast majority (Table 5).

**Table 4.** Quality of successful home sleep studies (*N* = 851)

Study quality	Number (%) of studies with a specific quality rating									
	All sites		Site 1		Site 2		Site 3		Site 4	
Excellent*	616	(72.4)	168	(69.1)	150	(79.4)	143	(71.9)	155	(70.5)
Very good^†^	118	(13.9)	47	(19.3)	17	(9.0)	24	(12.1)	30	(13.6)
Good^‡^	76	(8.9)	20	(8.2)	15	(7.9)	19	(9.6)	22	(10.0)
Fair^§^	41	(4.8)	8	(3.3)	7	(3.7)	13	(6.5)	13	(5.9)

*EEG, oximetry, airflow, chest wall, and abdomen signal: all ≥5 h.

^†^EEG, oximetry, airflow, and either chest wall or abdomen signal: all ≥ 4 h with at least one signal 4–5 h.

^‡^EEG, oximetry, airflow, and either chest wall or abdomen signal: all ≥ 3 h with at least one signal 3–4 h.

^§^Same as ‡, except that EEG was scored as sleep versus wake and not for sleep stages (N1, N2, N3, and REM).

**Table 5.** Percentage of successful home sleep studies with specified duration of artifact-free signal

Hours of artifact-free signal	EEG^†^	EOG^‡^	ECG^§^	Oximetry^‖^	Nasal pressure^¶^	Chest^#^	Abdomen^**^
≥6.0	81.8*	82.4	74.6	83.3	68.7	85.0	85.0
5.0–5.9	8.3	8.1	7.6	7.5	8.0	7.3	7.3
4.0–4.9	5.5	4.8	4.6	5.1	6.6	4.2	4.0
3.0–3.9	3.2	3.5	2.8	3.1	2.8	2.2	2.4
<3.0	1.2	1.2	10.4	1.0	13.9	1.3	1.3

*Percentage of successful sleep study (*N* = 851) for which the specified duration of artifact-free signal was obtained.

^†^Electroencephalogram.

^‡^Electrooculogram.

^§^Electrocardiogram.

^‖^Pulse oximetry.

^¶^Nasal pressure airflow signal.

^#^Chest wall movement signal.

^**^Abdominal movement signal.

### Reliability of scoring sleep, arousals, and sleep-disordered breathing events

To assess reliability of staging sleep and breathing abnormalities, 45 (5.3%) of the sleep studies were rescored by a second trained technician. Table 6 displays interscorer reliability metrics for total sleep time, wake after sleep onset, sleep latency, arousal frequency, and AHI, along with the percentages of sleep stages. Interscorer reliability was greatest for total sleep time (ICC: 0.96) and sleep latency (ICC: 0.95). For the arousal index, reliability was high for arousals during nonrapid eye movement (NREM) sleep (ICC: 0.90) or total sleep (ICC: 0.89), but lower for arousals during REM sleep (ICC: 0.68). Because the percentages of stage N3 sleep in the selected studies were too low to be examined for interscorer reliability (mean 2.7%, interquartile range 0.1%–3.3%), the three NREM stages (N1, N2, and N3) were combined. Reliability for percentages of NREM sleep (ICC: 0.94 [0.90–0.97]) and REM sleep (ICC: 0.95 [0.91–0.97]) was excellent. Finally, the reliability of AHI measurements was also extremely high (ICC > 0.98), regardless of whether hypopnea was defined by the 4% oxyhemoglobin desaturation criterion or by the 3% desaturation or arousal criteria. Comparisons of studies that were rescored to those that were not showed no significant differences in total sleep time, sleep latency, percentage of NREM sleep, the overall arousal index, NREM arousals index and the AHI values using either the 3% or 4% desaturation criteria (Supplementary Table S2). There were minor differences in sleep efficiency, percentage of NREM sleep, and the arousal index during REM sleep.

**Table 6.** Interscorer reliability metrics for sleep architecture, arousals, and AHI

Parameter	ICC	95% CI
Total sleep time, min	0.96	(0.94–0.98)
Sleep latency, min	0.95	(0.91–0.97)
WASO, min	0.86	(0.77–0.92)
NREM sleep, %*	0.94	(0.89–0.96)
REM sleep,%*	0.94	(0.89–0.96)
Arousals/h		
All sleep	0.89	(0.82–0.94)
NREM sleep	0.90	(0.83–0.94)
REM sleep	0.68	(0.51–0.82)
AHI, events/h		
4% desaturation	0.99	(0.98–1.00)
3% desaturation or arousal	0.98	(0.97–0.99)

WASO: wake after sleep onset defined as time awake after first onset of sleep.

*Percentage of total sleep time.

### Generalizability of the recruited sample

Table 7 shows the demographic and other health behavior characteristics of the 960 MACS participants who enrolled in this study and the 950 active MACS participants who did not. In general, the men who participated reflected the overall characteristics of the active MACS population. Statistically significant, but small, differences between the two groups were noted in age, BMI, race, smoking status, and alcohol use. The enrolled sample also had a higher proportion of HIV seropositive men (56.5% vs. 49.5%; *p* < 0.0025).

**Table 7.** Sample characteristics of MACS participants enrolled and not enrolled in the sleep study

	Enrolled (*N* = 960)	Not enrolled (*N* = 950)	*p*
Age, years	59 (15)	62 (14)	<0.0001
BMI, kg/m^2^	26.5 (6.5)	25.9 (6.1)	0.0021
Race			<0.0001
White	593 (61.8%)	701 (73.8%)	
Black	270 (28.1%)	172 (18.1%)	
Other	50 (5.2%)	46 (4.8%)	
Multiracial	46 (4.8%)	30 (3.2%)	
Missing data	1 (0.1%)	1 (0.1%)	
Ethnicity			0.20
Hispanic	126 (13.1%)	106 (11.2%)	
Non-Hispanic	834 (86.9%)	844 (88.8%)	
Education			0.23
≤11th grade	54 (5.6%)	48 (5.0%)	
12th grade	133 (13.9%)	100 (10.5%)	
Some college	249 (25.9%)	260 (27.4%)	
College degree	200 (20.8%)	203 (21.4%)	
Graduate work	327 (33.8%)	339 (35.7%)	
Missing data	0 (0.0%)	0 (0.0%)	
Income, $			0.25
<10 000	190 (19.8%)	162 (17.1%)	
10 000–19 000	132 (13.8%)	108 (11.4%)	
20 000–39 999	191 (19.9%)	201 (21.1%)	
40 000–59 999	144 (15.0%)	144 (15.1%)	
60 000–99 999	149 (15.5%)	169 (17.8%)	
≥100 000	152 (15.8%)	166 (17.5%)	
Missing data	2 (0.2%)	0 (0.0%)	
Smoking			0.02
Never	300 (31.3%)	280 (29.5%)	
Former	444 (46.2%)	498 (52.4%)	
Current	183 (19.7%)	155 (16.3%)	
Missing data	27 (2.8%)	17 (1.8%)	
Alcohol, drinks/week			0.045
None	212 (22.1%)	187 (19.7%)	
1–3	449 (46.8%)	413 (43.5%)	
≥4	263 (27.4%)	298 (31.3%)	
Missing data	36 (3.7%)	52 (5.5%)	
Cocaine use			0.14
No	847 (88.2%)	857 (90.2%)	
Yes	105 (11.0%)	81 (8.5%)	
Missing data	8 (0.8%)	12 (1.3%)	
HIV seropositive	542 (56.5%)	470 (49.5%)	0.0025

## Discussion

This study describes methods developed by the MACS for collection of home self-applied polysomnography, which included the EEG, to identify potentially sleep and breathing abnormalities in people living with and without HIV infection. The methods developed included well-defined participant instructions for using the Nox A1 recorder that ensured accurate placement of sensors without creating excessive discomfort or inconvenience. The analytic needs also dictated the development of rigorous approaches for central processing and scoring of the sleep data to characterize sleep architecture and breathing abnormalities. In addition, the quality of home sleep recordings was optimized by use of multiple levels of training and certification procedures for study staff, with close interactions among local sites, a central reading center, and a central coordinating center. Industry cooperation was also needed to address technical problems that were initially encountered with poor oximetry cable that was addressed by replacing the sensor with a redesigned one that was longer with better fit on the finger. Furthermore, initial concerns regarding subject burden incurred by recording the EEG were allayed with development of procedures for securing EEG electrodes using a front montage cable with self-applied electrodes. Recordings in over 900 participants confirmed that the EEG and EOG signals could be acquired without significant duration of artifact in these signals.

Not surprisingly, much of the variability in study quality was due to technical experience and equipment problems and varied by study site. Signals that posed the greatest challenge were the nasal pressure and ECG signals, as these were prone to artifact or loss of the sensor during the night. Inadequate placement and securing of the nasal cannula and the chest ECG leads was the most common reason for artifacts and sensor loss. Use of adhesive tape to secure the nasal cannula and proper cleaning of the skin for the ECG leads can mitigate loss of these signals. Reproducibility of staging of sleep and scoring of disordered breathing events was excellent for all parameters except for REM sleep, which may have been due, at least in part, to the lack of a chin sensor which is used in laboratory polysomnography.

As a result of these efforts, successful and reliable recordings were collected from approximately 85% of the participants on the first attempt, and 88.6% overall after two attempts. The quality of EEG data collected allowed scoring of the sleep–wake state and accurate delineation of NREM (N1, N2, and N3) and REM sleep stages. Scoring of arousals and identification of disordered breathing events was also possible, because EEG, oximetry, and measurements of airflow and chest and abdominal movement were available, as they would have been in an in-lab study. These methods can be applied to other community or population-based studies to advance our understanding of the effect of chronic conditions on sleep health and its influence on clinical outcomes.

The major contribution of the current study is that multiple levels of detailed procedures were established for collecting high-quality polysomnography data in the home. Prior studies such as the Sleep Heart Health Study [14, 15] and the SWAN study [16] have also employed unattended full montage polysomnography, but these studies required that two research staff travel to the participant’s home to do the initial hookup of the recording equipment. In addition, after completion of the sleep study, a staff member was needed to pick up the equipment and return it to the study site. In the current study, the use of clearly defined methods for self-application of the electrodes including the EEG, rigorous technician training on instructing participants, timely and consistent methods for evaluating the data quality, and regular reporting of study quality for troubleshooting all reinforced adherence to a common protocol. Despite the major differences in the collection and analysis between the current study and the Sleep Heart Health [14, 15] or the SWAN studies [16], rates of obtaining successful results in the studies were similar: 94.7% of participants in Sleep Heart Health [15] with three attempts, and in the present study 88.6% with only two attempts. Interestingly, the proportion of successful studies classified as being of good, very good, or excellent quality in the current study was 95.2%, versus 87.0% in the Sleep Heart Health Study [15]. Thus, the methods established should be of value for future research that require collection of home polysomnography with EEG without the need to send trained technicians to the home, which is cost-prohibitive. The approach to collection of unattended sleep data outlined here is also relevant for clinical practice, which is increasingly utilizing home testing, but for the most part without the inclusion of the EEG signal.

In the current study, a lower likelihood of a successful sleep study was associated with black race, cigarette smoking, and cocaine use. These factors may reflect differences in housing density, neighborhood sleep environment, or other factors which were not assessed and therefore could not be accounted for. Moreover, while adjustments were made for education and income level, it is possible that these adjustments did not fully account for the effects of a lower socioeconomic status. These issues notwithstanding, it should be noted that home self-applied polysomnography in these groups still had a high success rate and thus can still be utilized in these subsets of the population. Finally, in contrast to a previous report that age and BMI were weakly associated with the likelihood of obtaining a successful home unattended polysomnogram [17], the current study did not find such associations with age, BMI, educational level, income, or prevalent comorbidity. It is possible that lack of these associations could reflect the relatively small number of unsuccessful studies. In summary, the results of this study demonstrate that conducting self-applied polysomnography in the home is feasible and can be accomplished with a high success rate. Awareness of characteristics that are associated with an unsuccessful sleep study at home can allow for whether sleep testing in a laboratory setting should be used in specific subgroups.

## Supplementary Material

zpac011_suppl_Supplementary_Material
