# Supplementary material for: Methods for home-based self-applied polysomnography: the Multicenter AIDS Cohort Study
Source: Sleep Adv. 2022 Apr 29;3(1):zpac011. doi: 10.1093/sleepadvances/zpac011 (PMC9119085; doi:10.1093/sleepadvances/zpac011)
Supplement: zpac011_suppl_Supplementary_Material [file zpac011_suppl_supplementary_material.docx]

**ONLINE SUPPLEMENT: SUPPLEMENTARY TABLES AND FIGURES**

**Methods for Home-based Self-Applied Polysomnography:**

**The Multicenter AIDS Cohort Study**

Naresh M. Punjabi^1^, Todd Brown^2^, R Nisha Aurora^3^, Sanjay Patel^4^,

Valentina Stosor^5^, Joshua H. Cho^6^, Halla Helgadóttir^7^, Jón Skírnir Ágústsson^7^,

Gypsyamber D’Souza^8^, and Joseph B. Margolick^8^

^1^University of Miami, Miller School of Medicine; ^2^Johns Hopkins University, School of Medicine ^3^Robert Wood Johnson University Hospital, ^4^University of Pittsburgh, School of Medicine; ^5^Northwestern University Fienberg School of Medicine,

^6^University of California (Los Angeles), David Geffen School of Medicine, ^7^Nox Medical;

^8^Johns Hopkins University, Bloomberg School of Public Health

**Funding Information:** Supported by the following NIH grants: U01-HL146241; U01-HL146201; U01-HL146204; U01-HL146202; U01-HL146193; U01-HL146245, U01-HL146240; U01-HL146242; U01-HL146333; U01-HL146205; U01-HL146203; U01-HL146208; U01-HL146192; U01-HL146194. The data collection for the MACS/WIHS Combined Cohort Study was also supported by UL1-TR000004 (UCSF CTSA), UL1-TR003098 (JHU ICTR), UL1-TR001881 (UCLA CTSI), P30-AI-050409 (Atlanta CFAR), P30-AI-073961 (Miami CFAR), P30-AI-050410 (UNC CFAR), P30-AI-027767 (UAB CFAR), and P30-MH-116867 (Miami CHARM).

**Corresponding Author:**

Naresh M. Punjabi, MD, PhD

Division of Pulmonary, Critical Care, and Sleep Medicine

University of Miami, Miller School of Medicine

1600 NW 10^th^ Avenue

Miami, FL 33136

Phone: (305) 243-6388

Email: [npunjabi@miami.edu](mailto:npunjabi@miami.edu)

**Table S1:** Reasons for an unsuccessful home sleep study by month

| Study Month | Studies  Attempted | Studies Unsuccessful |  | Reason for unsuccessful study | | | | | | | | | | | |
| --- | --- | --- | --- | --- | --- | --- | --- | --- | --- | --- | --- | --- | --- | --- | --- |
|  |  |  |  | Oximetry  < 3 hours | |  | Impedance bands  < 3 hours | |  | | EEG  < 3 hours | |  | More than one signal  < 3 hours | |
| 1 | 60 | 9 |  | 4 | (44.4) |  | 3 | (33.3) | |  | 1 | (11.1) |  | 1 | (11.1) |
| 2 | 104 | 12 |  | 5 | (41.7) |  | 3 | (25.0) | |  | 3 | (25.0) |  | 1 | (8.3) |
| 3 | 91 | 14 |  | 4 | (28.6) |  | 7 | (50.0) | |  | 2 | (14.3) |  | 1 | (7.1) |
| 4 | 113 | 14 |  | 1 | (7.1) |  | 3 | (21.4) | |  | 7 | (50.0) |  | 3 | (21.4) |
| 5 | 110 | 12 |  | 4 | (33.3) |  | 2 | (16.7) | |  | 3 | (25.0) |  | 3 | (25.0) |
| 6 | 84 | 13 |  | 5 | (38.5) |  | 2 | (15.4) | |  | 3 | (23.1) |  | 3 | (23.1) |
| 7 | 88 | 17 |  | 7 | (41.2) |  | 2 | (11.8) | |  | 4 | (23.5) |  | 4 | (23.5) |
| 8 | 76 | 14 |  | 9 | (64.3) |  | 0 | (0.0) | |  | 4 | (28.6) |  | 1 | (7.1) |
| 9 | 64 | 17 |  | 3 | (17.6) |  | 5 | (29.4) | |  | 7 | (41.2) |  | 2 | (11.8) |
| 10 | 70 | 12 |  | 4 | (33.3) |  | 4 | (33.3) | |  | 3 | (25.0) |  | 1 | (8.3) |
| 11 | 49 | 10 |  | 2 | (20.0) |  | 2 | (20.0) | |  | 3 | (30.0) |  | 3 | (30.0) |
| 12 | 49 | 8 |  | 3 | (37.5) |  | 1 | (12.5) | |  | 3 | (37.5) |  | 1 | (12.5) |
| 13 | 31 | 7 |  | 4 | (57.1) |  | 0 | (0.0) | |  | 2 | (28.6) |  | 1 | (14.3) |
| 14 | 24 | 3 |  | 1 | (33.3) |  | 0 | (0.0) | |  | 1 | (33.3) |  | 1 | (33.3) |

Values reported are number of studies (%)

**Table S2:** Comparisons of sleep studies included and not included in the assessment of inter-scorer reliability

| Parameter | Not Included | |  | Included | |  |  |
| --- | --- | --- | --- | --- | --- | --- | --- |
|  | N=806 | |  | N=45 | |  |  |
|  | Mean | (SD) |  | Mean | SD |  | p-value |
| Total sleep time, min | 371.7 | (90.6) |  | 395.3 | (77.3) |  | 0.08 |
| Sleep latency, min | 18.8 | (25.8 |  | 21.7 | (25.5) |  | 0.47 |
| Sleep efficiency, % | 84.6 | (11.5) |  | 88.2 | (8.9) |  | 0.03 |
|  |  |  |  |  |  |  |  |
| NREM sleep, % | 77.0 | (9.7) |  | 81.1 | (9.0) |  | <0.01 |
| REM sleep, % | 12.5 | (6.6) |  | 12.2 | (7.8 |  | 0.76 |
|  |  |  |  |  |  |  |  |
| Arousals/hour |  |  |  |  |  |  |  |
| All sleep | 15.7 | (9.3) |  | 13.4 | (8.7) |  | 0.11 |
| NREM sleep | 16.8 | (10.1) |  | 14.5 | (9.9) |  | 0.14 |
| REM sleep | 8.6 | (8.1) |  | 6.0 | (4.5) |  | 0.02 |
|  |  |  |  |  |  |  |  |
| AHI, events/hr |  |  |  |  |  |  |  |
| 4% desaturation | 11.1 | (14.4) |  | 10.1 | (12.1) |  | 0.72 |
| 3% desaturation | 15.8 | (16.3) |  | 14.9 | (14.6) |  | 0.63 |

**Figure S1**: Number of home sleep studies and proportion successful, by study month.

**Figure S2:** Percent of studies rated as excellent, very good, good, or fair quality by study month and site.
